# Hypertension reduces soluble guanylyl cyclase expression in the mouse aorta via the Notch signaling pathway

**DOI:** 10.1038/s41598-017-01392-1

**Published:** 2017-05-02

**Authors:** Catarina Rippe, Baoyi Zhu, Katarzyna K. Krawczyk, Ed. Van Bavel, Sebastian Albinsson, Jonas Sjölund, Erik N. T. P. Bakker, Karl Swärd

**Affiliations:** 1 Department of Experimental Medical Science, Lund University, Lund, Sweden; 2 Department of Biomedical Engineering and Physics, Academic Medical Center, Amsterdam, The Netherlands; 3 Department of Laboratory Medicine, Lund University, Lund, Sweden

## Abstract

Hypertension is a dominating risk factor for cardiovascular disease. To characterize the genomic response to hypertension, we administered vehicle or angiotensin II to mice and performed gene expression analyses. AngII treatment resulted in a robust increase in blood pressure and altered expression of 235 genes in the aorta, including *Gucy1a3* and *Gucy1b3* which encode subunits of soluble guanylyl cyclase (sGC). Western blotting and immunohistochemistry confirmed repression of sGC associated with curtailed relaxation via sGC activation. Analysis of transcription factor binding motifs in promoters of differentially expressed genes identified enrichment of motifs for RBPJ, a component of the Notch signaling pathway, and the Notch coactivators FRYL and MAML2 were reduced. Gain and loss of function experiments demonstrated that JAG/NOTCH signaling controls sGC expression together with MAML2 and FRYL. Reduced expression of sGC, correlating with differential expression of MAML2, in stroke prone and spontaneously hypertensive rats was also seen, and RNA-Seq data demonstrated correlations between *JAG1*, *NOTCH3*, *MAML2* and *FRYL* and the sGC subunits *GUCY1A3* and *GUCY1B3* in human coronary artery. Notch signaling thus provides a constitutive drive on expression of the major nitric oxide receptor (GUCY1A3/GUCY1B3) in arteries from mice, rats, and humans, and this control mechanism is disturbed in hypertension.

## Introduction

Despite a broad repertoire of pharmacological therapies to normalize an elevated blood pressure, hypertension currently represents the most prevalent risk factor for cardiovascular morbidity and mortality worldwide. Hypertension accelerates vascular retention of atherogenic lipoproteins^1^ and promotes inflammation^2^, but it also impairs nitric oxide (NO) mediated vasodilatation^3^. Disruption of NO-mediated vasodilatation is detrimental for blood flow regulation^4^ and is considered to represent a long-term pathogenic mechanism for several clinical manifestations, such as stroke and myocardial infarction, of a chronically raised blood pressure^3, 5^. Reduction of NO-dependent vasodilatation in hypertension is due in part to a reduction of the protein level of soluble guanylyl cyclase (sGC)^6, 7^, the major NO receptor in the vascular wall.

sGC is a protein complex consisting of two subunits derived from the genes *GUCY1A3* and *GUCY1B3*, that generates cyclic GMP in response to NO in vascular smooth muscle cells^8^. Genetic studies have demonstrated that intronic sequence variants in *GUCY1A3* associate with hypertension^9^ and coronary heart disease^10^, underscoring the relevance of sGC both as a cause and as an effector of cardiovascular disease. Familial mutations in *GUCY1A3* moreover predispose for myocardial infarction^11^ and brain vasculopathy^12^. Transcriptional control mechanisms for *GUCY1A3* and *GUCY1B3* therefore represent a medically prioritized area of investigation that may uncover novel targets for therapy of cardiovascular disease.

A breakthrough was recently made in regard to transcriptional regulation of sGC when it was shown that *Gucy1a3* and *Gucy1b3* are controlled by conserved binding motifs for RBPJ, a core component of the Notch signaling pathway, during cardiac valve development^13^. The Notch signaling pathway is activated by cell surface receptors (NOTCH1-4) that are responding to cell attached ligands (JAG1, JAG2, DLL1, DLL3 and DLL4), and this results in the release of the intracellular domain of the receptor through γ-secretase-dependent cleavage^14^. The intracellular domain of the receptor then enters the nucleus where it activates transcription together with RBPJ and numerous coactivators, including proteins from the mastermind like (MAML) coactivator family and others, such as FRYL^15^. Notch signaling is known to play a fundamental role in vascular development^16^, and a regulatory role of NOTCH in the blood vessel wall beyond embryonic development is indicated by the fact that postnatal deletion of RBPJ in smooth muscle causes profound structural and molecular changes^17^.

In the current study we surveyed the impact of hypertension on gene activity in the mouse aorta and demonstrate compelling repression of the sGC subunits GUCY1A3 and GUCY1B3. Bioinformatic analyses pointed to the involvement of RBPJ-binding motifs, and we therefore addressed the hypothesis that Notch signaling controls the expression of sGC in vascular smooth muscle. Using both gain and loss of function approaches we demonstrate that Notch signaling represents a powerful control mechanism for sGC expression in mouse and human vascular smooth muscle cells.

## Results

**Figure 1:**
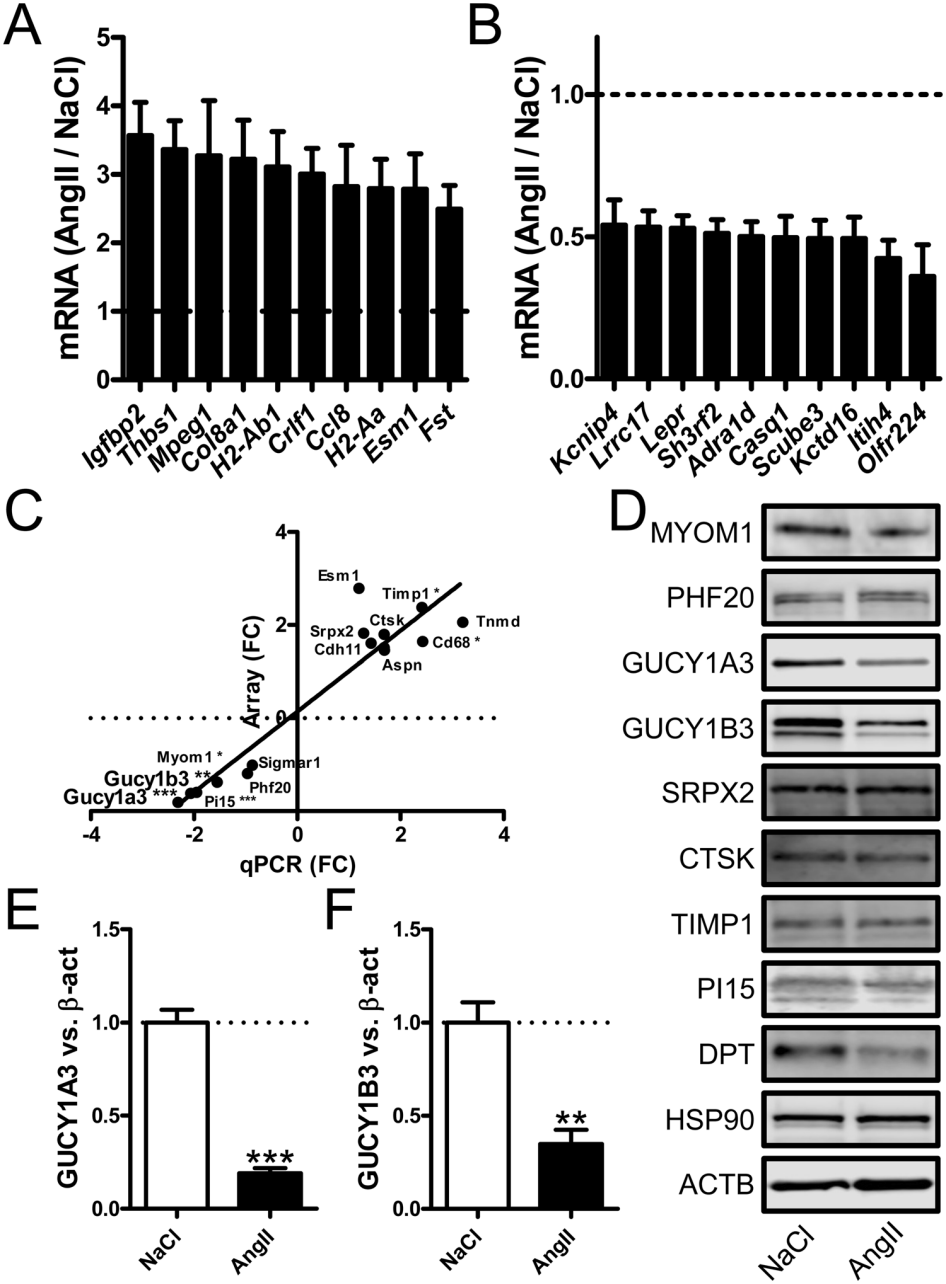
Differentially expressed mRNAs in the hypertensive aorta as determined by microarray analysis. To identify differentially expressed genes in the hypertensive arterial wall, we isolated the aorta from vehicle (physiological saline or NaCl, n = 7, mean arterial blood pressure (MAP): 103 ± 11 mmHg) and angiotensin II (AngII)-treated mice (n = 8, MAP: 186 ± 6 mmHg) and subjected the isolated RNA to microarray analysis. 235 genes were differentially expressed (SAM analysis, Q = 0). Panels A and B show the ten most upregulated and the ten most repressed mRNAs, respectively. All loading controls used herein for western blotting changed less than 10%, and non-significantly (Hsp90: q = 11; Gapdh: q = 51; Actb: q = 35), in the array experiment. 14 genes were chosen for confirmation by RT-qPCR in a material that included the original samples plus four samples in each group that were not included in the array experiment. Six of the mRNA changes, including those for *Gucy1a3* and *Gucy1b3* could be confirmed (*P < 0.05, **P < 0.01, P < 0.001). In panel C the fold change in the qRT-PCR experiment is plotted versus the fold change in the array experiment. A highly significant correlation (Spearman method, P < 0.0001) was seen. Western blotting was done in (**D**) using aortic protein lysates (n = 6/group) to assess changes for a selection of the genes in (**C)**. To allow for detection of multiple targets, membranes were cut horizontally above and below the known molecular weights and hence full lane blots are not available. Panels E and F show summarized data at the protein level for the subunits (GUCY1A3, GUCY1B3) of the soluble guanylyl cyclase (sGC).

To uncover pathogenic mechanisms of hypertension in the vessel wall, we treated mice with Angiotensin II (AngII) or vehicle (physiological saline, NaCl) for three weeks followed by microarray analysis of mRNAs. We isolated RNA from the aorta in view of the sensitivity of this artery to atherosclerosis and because of its large biomass compared to other arteries. 235 mRNAs were differentially expressed at Q = 0. Figure 1A and B show the most highly induced and most highly repressed mRNAs. These mRNAs give rise to matrix constituents, membrane receptors, channel interacting proteins and signaling molecules. Based on predicted functional importance in the vascular wall, we chose 14 out of the 235 differentially expressed mRNAs for confirmation using qRT-PCR (Fig. 1C). Changes in the same direction were seen throughout, and the data sets correlated significantly (P < 0.0001). *Gucy1a3* and *Gucy1b3*, encoding subunits of soluble guanylyl cyclase, were repressed in the array experiment (Q = 0 for both) and in the confirmatory qRT-PCR experiment (P < 0.001 and P < 0.01, respectively, Fig. 1C). We next used western blotting (Fig. 1D) to examine changes at the protein level. GUCY1A3 and GUCY1B3 were reduced by 81 ± 3% (P < 0.001, Fig. 1D,E) and by 65 ± 8% (P < 0.01, Fig. 1D,F). Myomesin 1 (MYOM1) and Dermatopontin (DPT) were the only other targets among the nine chosen for western blotting that were significantly different at both the mRNA and at the protein level (Fig. 1C,D). We concluded that subunits of the soluble guanylyl cyclase (sGC) are compellingly repressed in the hypertensive mouse aorta.

**Figure 2:**
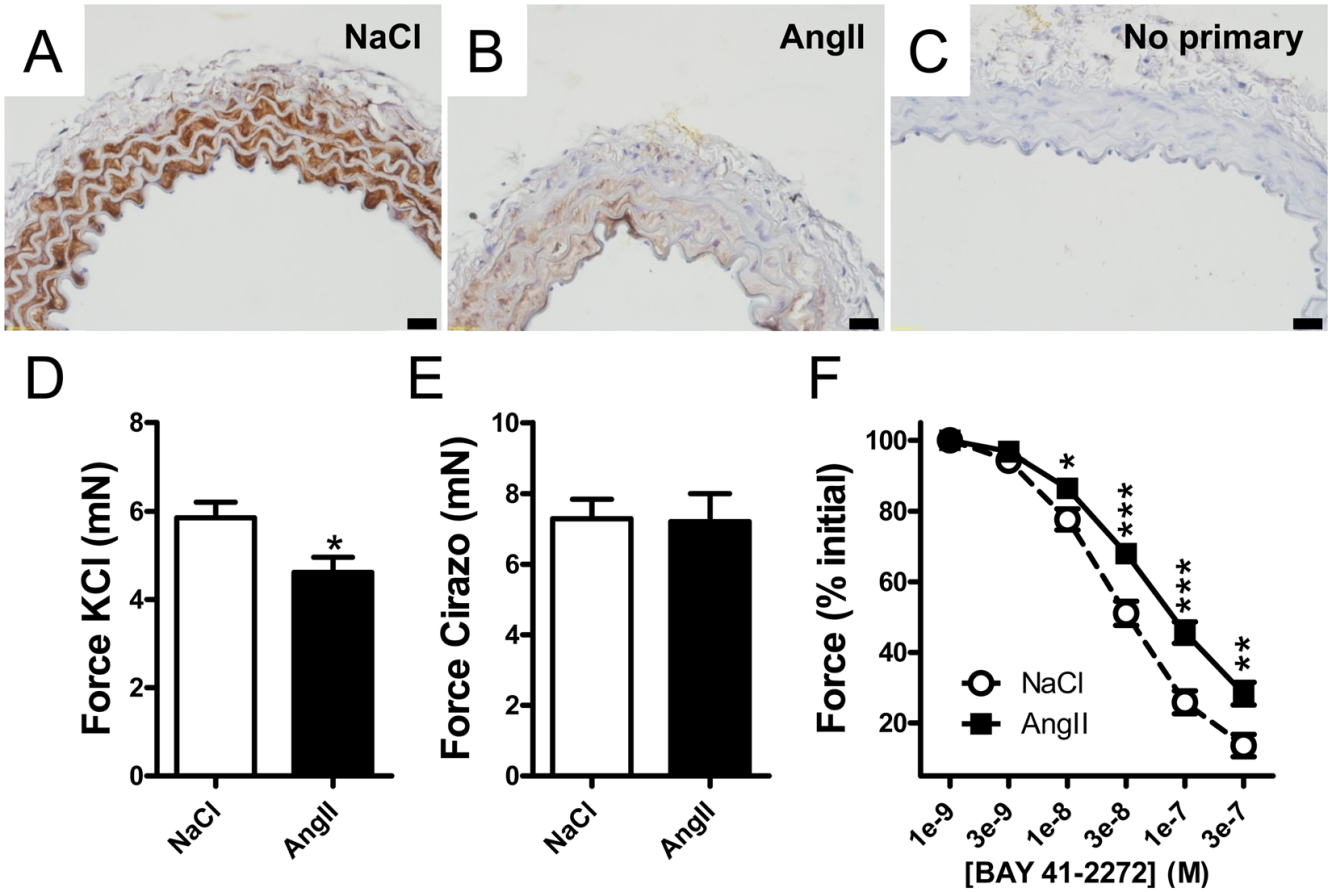
Reduced expression of sGC in the hypertensive aorta. The aorta from vehicle- and AngII-treated mice was isolated and fixed for immunohistochemistry. Panels A and B show staining for GUCY1B3 (brown) as indicated. Panel C represents a primary antibody omission control. Scale bars represent 20 µm. Aortae were also isolated and mounted for force measurements (n = 8/group) using wire myography. Depolarization-induced (60 mM KCl) contraction was reduced (**D**), but contraction in response to the α_1_-adrenergic agonist cirazoline was unchanged (**E**). In (**F)**, preparations were relaxed by sGC activation using BAY 41–2272 following pre-contraction with cirazoline. *P < 0.05, **P < 0.01, P < 0.001.

The tissue distribution of sGC was next examined by immunohistochemistry. Reduced staining for GUCY1B3 (brown) was seen in the hypertensive aortic media (Fig. 2A versus B). To establish if repression of sGC has a functional impact we next mounted aortic rings from vehicle- and AngII-treated mice in wire myographs. Depolarization-induced contraction was reduced after AngII treatment (Fig. 2D), but α_1_-receptor-induced (cirazoline) contractility was unchanged (Fig. 2E). Relaxation of the latter contraction by BAY 41–2272, a direct activator of sGC, was reduced at all concentrations exceeding 3 nM (Fig. 2F). This demonstrates that sGC repression in the hypertensive aorta has direct functional consequences.

**Figure 3:**
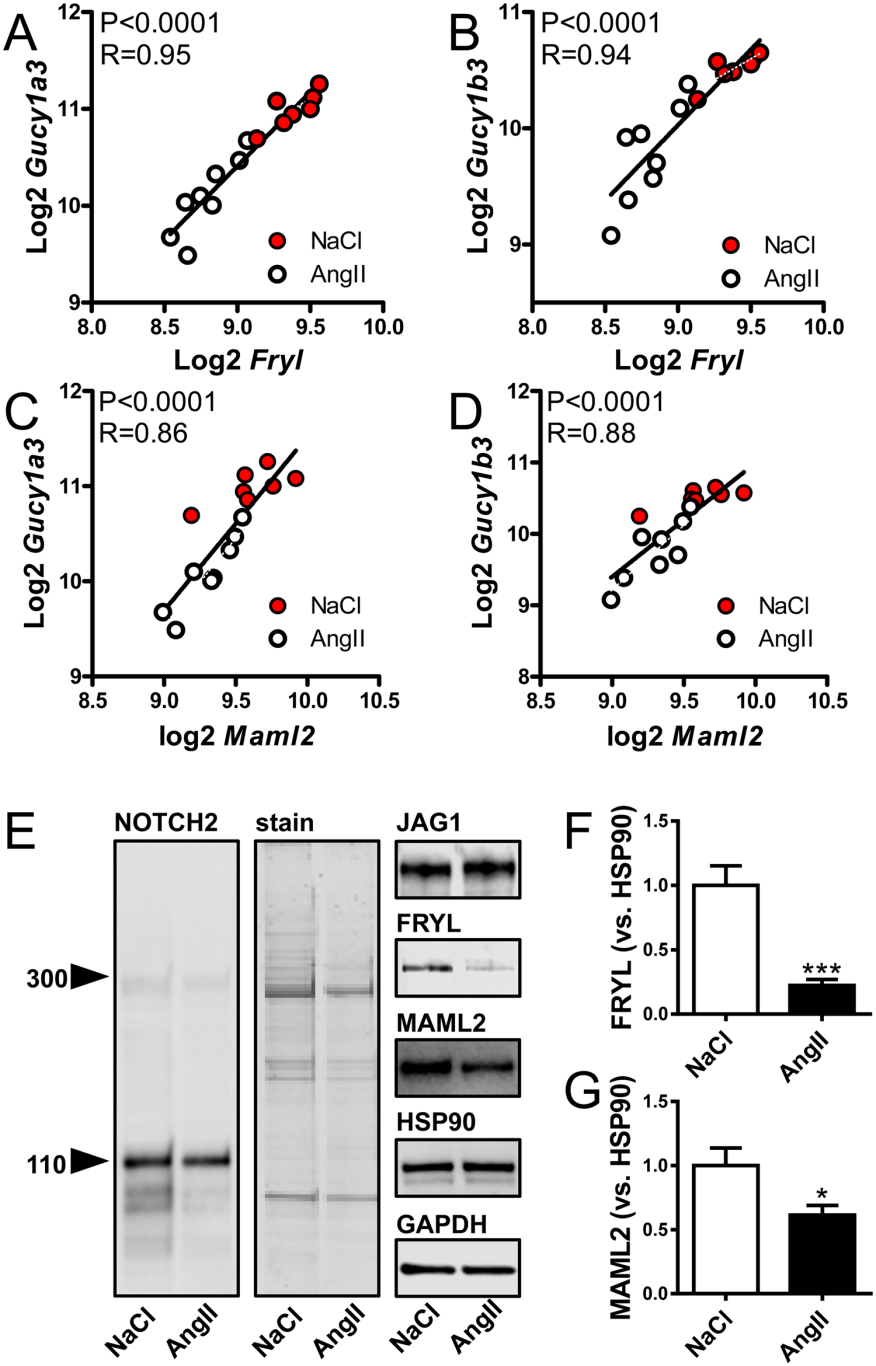
Repression of *Gucy1a3* and *Gucy1b3* correlates with changes in the Notch coactivators *Fryl* and *Maml2*. Panels A through D show correlations between *Fryl* and *Maml2* and the sGC subunits *Gucy1a3* and *Gucy1b3* at the mRNA level. Data is from the microarray experiment. P-values and Spearman Rho coefficients are given in the respective panels. Correlations were also observed when AngII-treated mice were considered individually (*Gucy1a3*: P_AngII_ = 0.005, P_vehicle_ = 0.06; *Gucy1b3*: P_AngII_ = 0.015, P_vehicle_ = 0.24). Panel E shows western blots (n = 6/group) for aortic NOTCH2, JAG1, FRYL and MAML2 in vehicle and AngII-infused mice. Coomassie-stained proteins remaining on the gel after transfer was used as loading control for NOTCH2. HSP90 and GAPDH were used as loading controls for JAG1, FRYL and MAML2. Panels F and G show summarized western blot data for FRYL and MAML2, respectively (versus HSP90, *P < 0.05).

To decipher the molecular basis of reduced sGC expression, we next used a bioinformatical method that determines enrichment of binding motifs for transcription factors in the promoters of differentially expressed genes (i.e. the Q = 0 gene list from the microarrays). This analysis revealed significant (P < 0.05) enrichment of 81 binding motifs (not shown). RBPJ (P = 0.01 for motif enrichment), a core component of the Notch signaling pathway, caught our attention because prior work demonstrated that GUCY1A3 and GUCY1B3 are controlled by NOTCH in the context of cardiac valve development^13^. We therefore cross-referenced our gene list against proteins that interact with NOTCH as defined by mass spectrometry^15^. This analysis identified reduced expression of two coactivators that contribute to gene activation by NOTCH: FRYL (Q = 0, P = 1.4 × 10^−5^) and MAML2 (Q = 2.6, P = 0.02). To support the hypothesis that changes in FRYL and MAML2 may be responsible for repression of GUCY1A3/GUCY1B3, we performed correlation analyses at the mRNA level. Both coactivators correlated with both sGC subunits (Fig. 3A–D). Similar correlations were seen when AngII-treated (white circles) mice were considered individually, ruling out any cluster effects. Taken together, these analyses established a link between reduced sGC expression in the hypertensive aortic wall and altered NOTCH signaling.

Using western blotting we next examined NOTCH2 which was present at a higher level than other NOTCH receptors in the mouse aorta as judged from the array signal (Supplementary Fig. 1). NOTCH2 was mostly cleaved (i.e. migrated at 110 kDa), but was unchanged in AngII-treated mice compared to controls (Fig. 3E). JAG1, a NOTCH receptor ligand, was also unchanged (Fig. 3E). Western blotting for FRYL revealed a major band just above 150 kDa that was drastically reduced (Fig. 3E,F). This molecular weight is consistent with exon usage in arteries (http://www.gtexportal.org), and a band at the same molecular weight was found to be reduced using another antibody (Supplementary Fig. 2). A single band was seen when blotting for MAML2, and this band was reduced by AngII treatment (Fig. 3E,G). These findings supported the possibility that hypertension may impair signaling downstream of the NOTCH receptor in the aorta.

**Figure 4:**
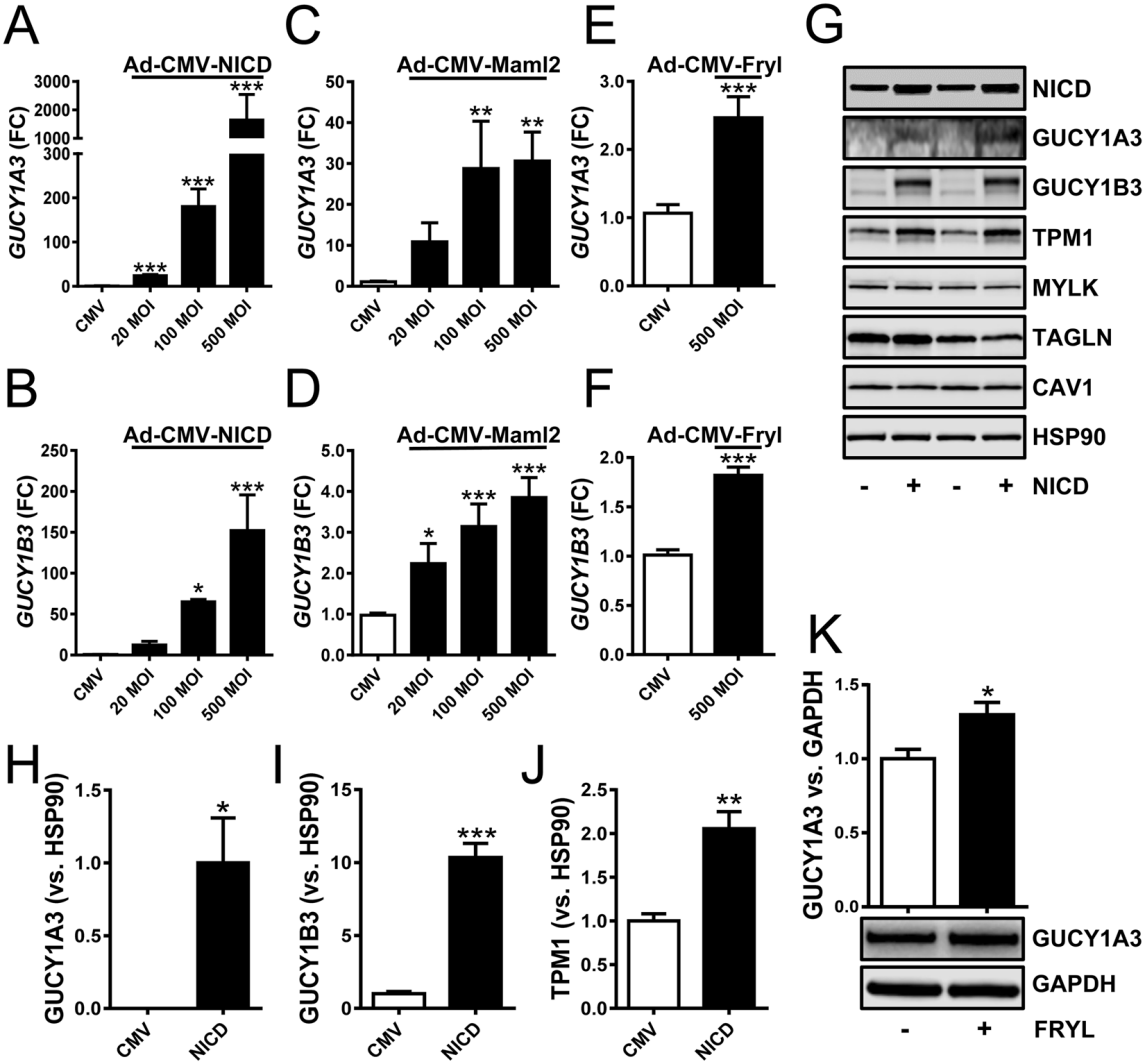
Adenoviral transduction of *NOTCH*, *MAML2* and *FRYL* increases sGC in smooth muscle cells. Human coronary artery smooth muscle cells were treated with Ad-CMV-null adenovirus (CMV) or adenoviruses for the intracellular domain of *NOTCH2* (Ad-CMV-NICD, (**A**,**B**), for full length *MAML2* (Ad-CMV-MAML2, (**C**,**D**)), and for a C-terminal fragment of *FRYL* (Ad-CMV-FRYL, (**E**,**F**)). The mRNA levels of *GUCY1A3* and *GUCY1B3* were then determined using qRT-PCR (FC: fold change, MOI: multiplicity of infection). In panel G GUCY1A3 and GUCY1B3 were determined by western blotting following NICD transduction. Compiled Western blot data is shown in the bar graphs in (**H**) through (**J**). Panel K shows an original western blot for GUCY1A3 following transduction of the *FRYL* C-terminus and, above, a bar graph with summarized data. *P < 0.05, **P < 0.01, P < 0.001.

We next tested if NOTCH controls sGC expression using smooth muscle cells from the human coronary artery. We used an adenovirus to overexpress the intracellular domain of NOTCH2 (NICD). Adenoviral transduction of NICD caused concentration-dependent induction of the guanylyl cyclases at the mRNA level, with 100–1000-fold changes at the highest titers (Fig. 4A,B). Overexpression of MAML2 had a similar effect, with changes ranging from 3–30-fold (Fig. 4C,D). We also overexpressed a fragment of FRYL containing domains known to be important for transcriptional activation^18^. This led to 2-fold induction of the sGC subunits at the mRNA level (Fig. 4E,F).

To determine whether NICD transduction increased the protein levels of GUCY1A3 and GUCY1B3 we again used western blotting (Fig. 4G through J). NICD was increased as expected (Fig. 4G, P < 0.001), and both GUCY1A3 and GUCY1B3 proteins increased in parallel (Fig. 4G and summarized data in H,I). We also examined a number of smooth muscle differentiation markers. With the exception for tropomyosin (TPM1), which increased (Fig. 4G,J), most differentiation markers, including myosin light chain kinase (MYLK), SM22α (TAGLN) and caveolin-1 (CAV1) were not affected by NICD overexpression (Fig. 4G). Overexpression of FRYL, finally, was associated with a small but significant increase of GUCY1A3 (Fig. 4K).

**Figure 5:**
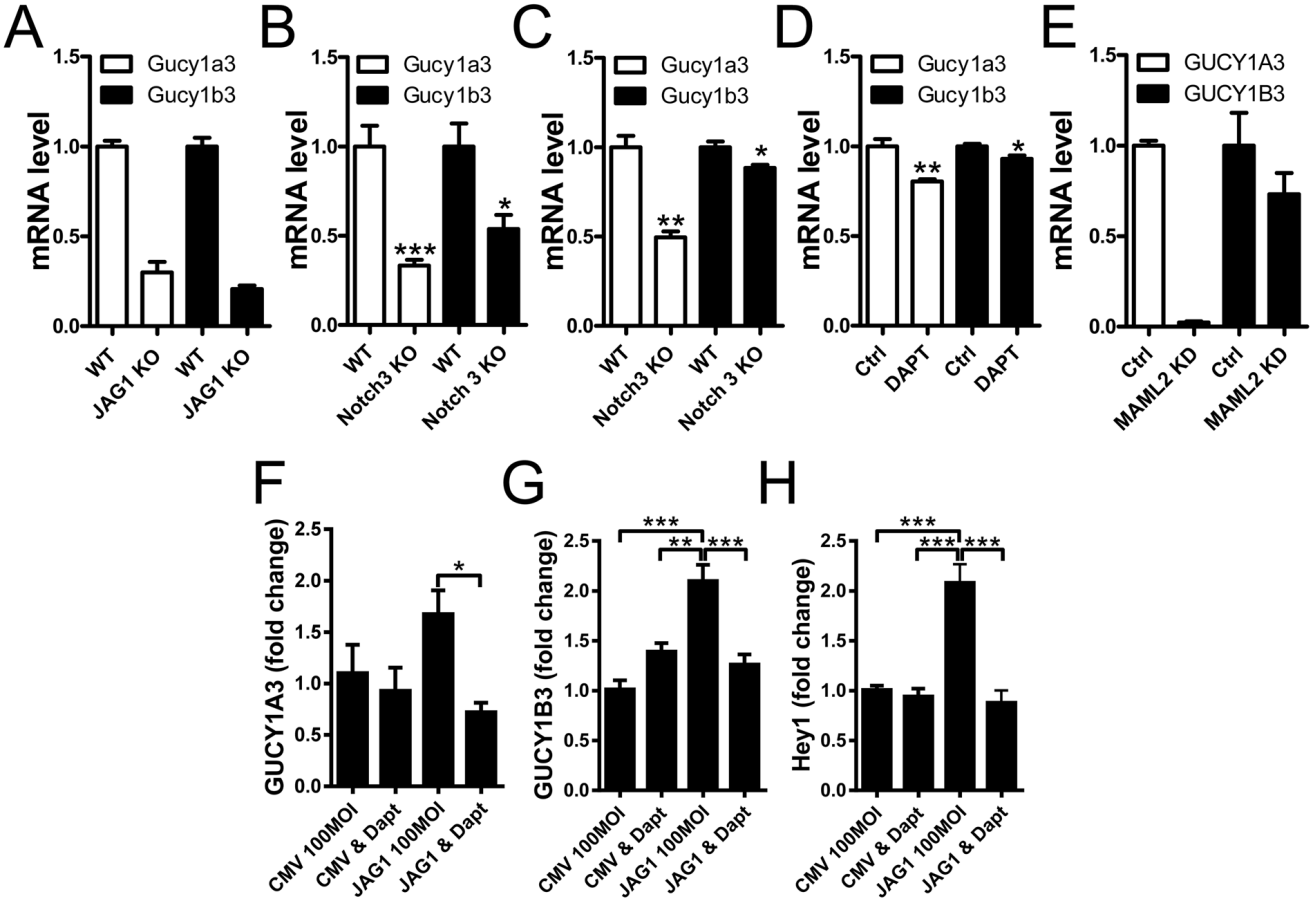
Genetic and pharmacological inhibition of Notch signaling leads to reduced *Gucy1a3*/*Gucy1b3* expression in smooth muscle. Transcriptomic data sets from knockout/knockdown models were analyzed for changes in sGC subunits. Panel A shows expression of sGC subunits in the 14.5 day embryonic aorta from *Jag1* wild type (WT) and knockout (KO) mice as determined by deep sequencing (n = 2, and hence no statistical analysis performed). Panel B shows sGC expression in brain microvascular fragments from WT and *Notch3* KO mice (n = 6), and panel C shows sGC expression in caudal arteries from *Notch3* WT and KO mice (n = 3), both determined by microarray analysis. Panel D shows sGC expression as assessed using deep sequencing of RNA from smooth muscle cells isolated from the mouse descending aorta and treated with γ-secretase inhibitor (DAPT). Panel E shows sGC expression in parotid adenocarcinoma cells following retroviral-based knockdown of MAML2 (n = 2). In panels F through H human coronary artery smooth muscle cells were incubated with the γ-secretase inhibitor DAPT following treatment with Ad-CMV-null and Ad-CMV-*JAG1*, respectively. sGC expression was determined using qRT-PCR. n = 6/group *P < 0.05, **P < 0.01, P < 0.001.

Having established that NOTCH/MAML2/FRYL are capable of driving sGC expression in smooth muscle cells using overexpression strategies we next sought loss of function data. Initial attempts to inhibit Notch signaling in cell culture using the γ-secretase inhibitor DAPT (N-[N-(3,5-Difluorophenacetyl)-L-alanyl]-S-phenylglycine t-butyl ester) were ineffective, possibly due to the low expression of guanylyl cyclases in cultured cells. We therefore analyzed publically available transcriptomic data sets from knockout/knockdown models. The first dataset was for aortic smooth muscle cells from E14.5 mouse embryos lacking JAG1 in smooth muscle (GSE60643)^19^. This RNA-Seq dataset contained two biological replicates, but repression of the two sGC subunits was very consistent (Fig. 5A). The next two datasets were for brain microvessels and caudal arteries from mice that lack NOTCH3^17, 20^. Sizeable repression of sGC subunits was found in both datasets (Fig. 5B,C). We also considered mouse aortic smooth muscle cells treated with γ-secretase inhibitor (DAPT)^19^ which revealed only small, albeit significant, effects on sGC (Fig. 5D). The small effect by DAPT is similar to our own findings in cell culture. The last dataset that we analyzed consisted of two biological replicates and evaluated the effect of MAML2 knockdown in parotid adenocarcinoma cells^21^. Here, clear-cut reduction of GUCY1A3, but a smaller effect on GUCY1B3 (Fig. 5E), was seen. We concluded that varied loss of function approaches supported the view that NOTCH signaling provides a driving force for sGC expression.

The finding in Fig. 5A that JAG1 knockout potently reduces sGCs^19^ inspired us to overexpress JAG1 in the human coronary artery smooth muscle cells followed by treatment with DAPT (10 µM) to inhibit NOTCH cleavage. Consistent with our pilot experiments, DAPT was without effect on sGC expression in control cells (Fig. 5F,G). Following overexpression of JAG1 on the other hand, DAPT significantly repressed sGC (Fig. 5F,G). Similar results were obtained for the prototypical NOTCH target gene *HEY1* (Fig. 5H). These findings supported our initial suspicion that NOTCH signaling is low in cultured smooth muscle cells *in vitro* and also demonstrated that increased JAG1 expression confers sensitivity of sGC expression to NOTCH inhibition.

**Figure 6:**
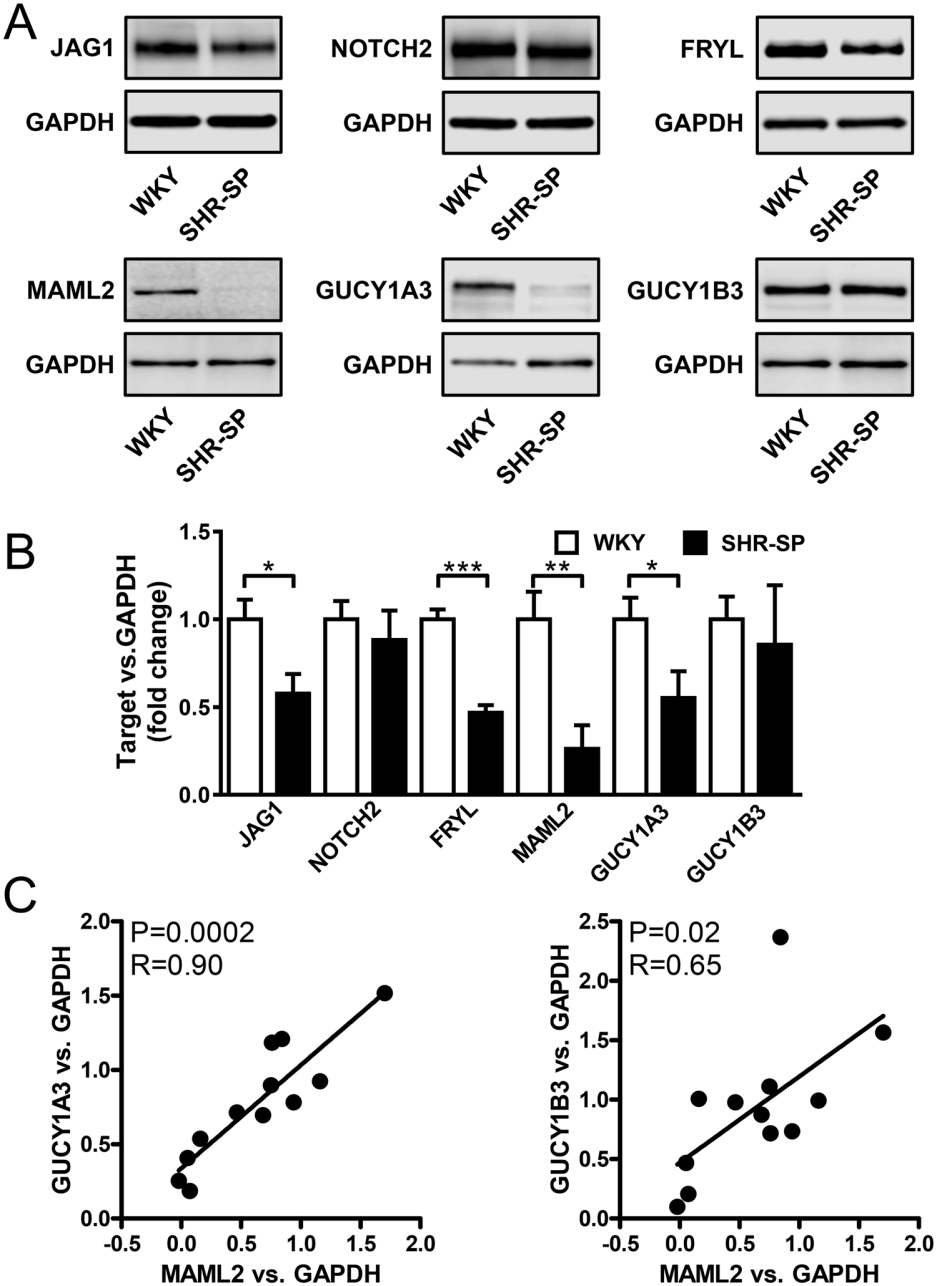
Repression of MAML2 and GUCY1A3 in the aorta of spontaneously hypertensive rats. Panel A shows western blots using aortae from Wistar Kyoto (WKY) and spontaneously hypertensive and stroke prone rats (SHR-SP) using the indicated antibodies. Bar graphs in panel B show summarized western blot data for JAG1, cleaved NOTCH2, FRYL, MAML2, GUCY1A3 and GUCY1B3. Panel C shows correlations at the protein level between MAML2 and GUCY1A3 and between MAML2 and GUCY1B3. n = 6/group, *P < 0.05, **P < 0.01.

Our findings so far argued that aortic sGC is repressed in AngII-induced hypertension and that this may be secondary to effects on NOTCH signaling. However, AngII is known to have effects beyond the elevation of blood pressure. To examine if NOTCH-mediated reduction of sGC occurred in other models of hypertension, we used spontaneously hypertensive and stroke prone rats (SHR-SP) and their normotensive counterpart, the WKY rat. Western blotting of aortic homogenates demonstrated that JAG1 and cleaved NOTCH2 were reduced and unchanged respectively (Fig. 6A,B). In keeping with our results in AngII hypertensive aorta, both FRYL and MAML2 were reduced at the protein level (Fig. 6A,B). GUCY1A3 expression was also reduced in SHR-SP compared to WKY, but GUCY1B3 expression was unchanged (Fig. 6A,B). The protein level of MAML2 nonetheless correlated with both GUCY1A3 and with GUCY1B3 (Fig. 6C).

**Figure 7:**
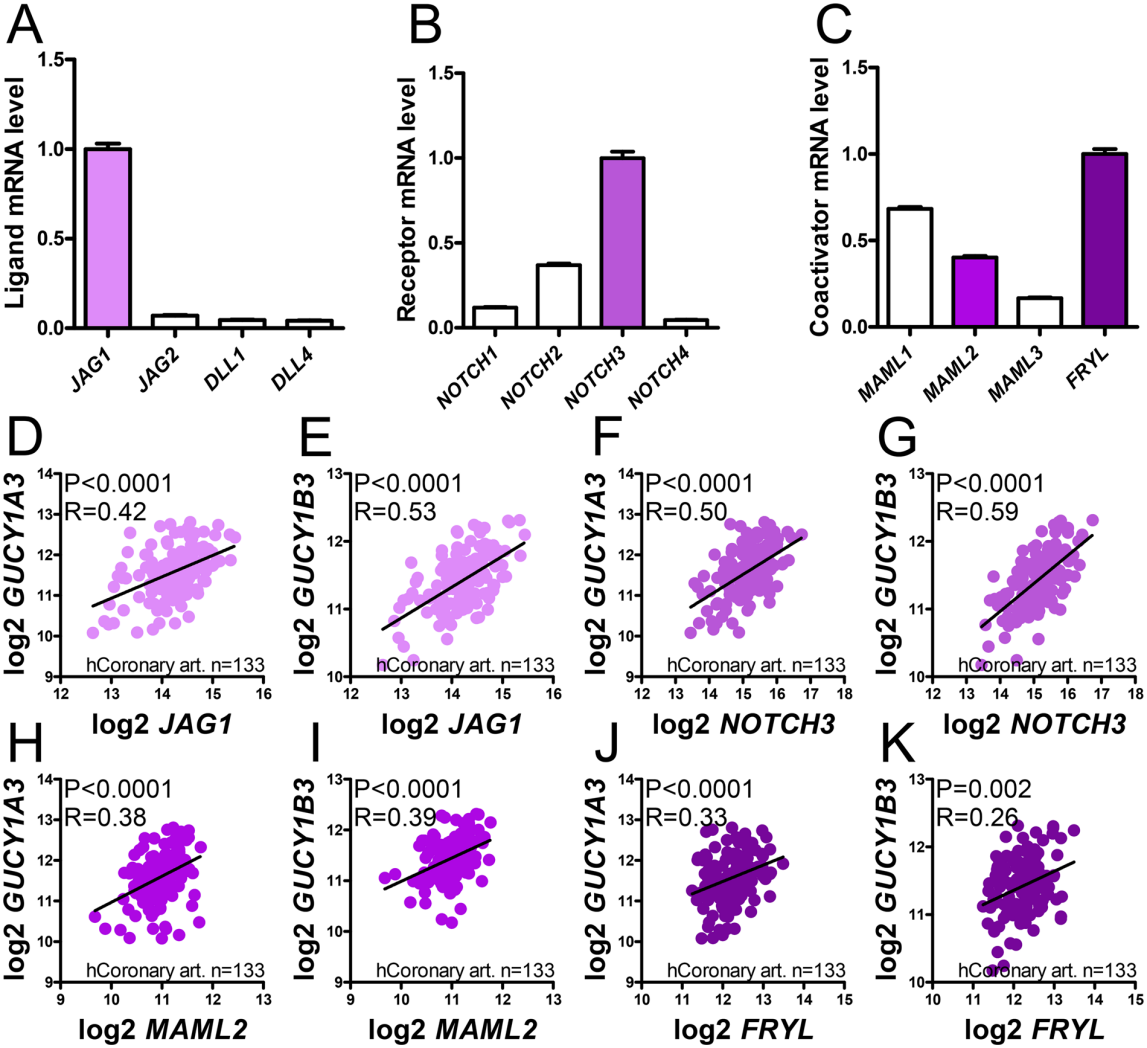
*JAG1*, *NOTCH3*, *MAML2* and *FRYL* mRNAs all correlate with *GUCY1A3* and *GUCY1B3* mRNAs in the human coronary artery. Expression data for the human coronary artery was downloaded from the GTExPortal.org and TMM normalized as described^34^. Panels A though C show relative expression levels for *NOTCH* ligands (**A**), receptors (**B**), and coactivators (**C**). Panels D through K show correlations for *JAG1*, *NOTCH3*, *MAML2*, and *FRYL* versus *GUCY1A3* and *GUCY1B3*. P-values and Spearman Rho coefficients are given in the respective panels.

Up to this point our findings supported the view that NOTCH signaling provides a constitutive drive for aortic sGC expression in the mouse aorta that is disturbed by hypertension via repression of the coactivators MAML2 and FRYL. To support that this signaling pathway drives sGC expression also in human arteries, we downloaded mRNA expression data from the GTExPortal. We first examined relative expression levels of NOTCH ligands, receptors, and coactivators in the coronary artery (Fig. 7A–C). *JAG1* was the most highly expressed NOTCH ligand (Fig. 7A). *NOTCH3* dominated among the receptors followed by *NOTCH2* (Fig. 7B). *FRYL* was most highly expressed among the coactivators considered, and *MAML2* levels were intermediate (Fig. 7C). Correlation analyses were next made to examine relationships between these mRNAs and sGC. *JAG1* correlated tightly with *GUCY1A3* and *GUCY1B3* (Fig. 7D,E). *NOTCH3* similarly correlated with *GUCY1A3* and with *GUCY1B3* (Fig. 7F,G), as did *NOTCH2* (P < 0.0001, not depicted), albeit with smaller correlation coefficients (R = 0.35 and 0.37). *MAML2* (Fig. 7H,I), but not *MAML1* (P = 0. 91 and 0.21, not depicted), moreover correlated with both sGC subunits. *FRYL*, finally, correlated with both *GUCY1A3* and *GUCY1B3* (Fig. 7J,K).

## Discussion

Results of the current study support the novel concept that Notch signaling provides a homeostatic drive for sGC expression in adult arteries that is inhibited by hypertension. The latter effect appears to be funneled via the Notch coactivators MAML2 and FRYL (also known as AF4P12)^15, 22^, whose reduction uncouples receptor cleavage from transcriptional activation of a subset of target genes. We find that both MAML2 and, albeit to a lesser extent, FRYL have the capacity to independently drive sGC expression, making them potential targets for prevention of cardiovascular disease caused by an elevated blood pressure. Previous work has shown that Notch signaling controls expression of sGC in the heart valves^13^ and in cancer cells^23^. We now demonstrate that this applies to the vascular wall where nitric oxide (NO)-cGMP signaling plays a fundamental physiological role via regulation of arterial resistance^4^. It is well documented that hypertension impairs endothelial dependent dilatation by reducing NO bioavailability in experimental animals^24, 25^ and humans^3, 5, 26^. Hypertension moreover decreases the arterial response to the nitric oxide-releasing drug sodium nitroprusside and reduces the expression of sGC^6, 7, 25, 27–30^, but the basis of the latter effect has remained unexplored. Our findings thus provide insight into a decade-long conundrum regarding the repression of sGC in hypertension by showing that this is due to perturbed Notch signaling downstream of the receptor.

Prior studies have demonstrated that mechanical forces acting on vascular smooth muscle influence Notch signaling. For example, *in vitro* exposure of rat vascular smooth muscle cells to cyclic strain was found to impair Notch signaling via repression of Notch ligands (JAG1) and receptors (NOTCH1, NOTCH3), leading to reduced RBPJ transcriptional activity^31^. In contrast, another study demonstrated that pulmonary arterial hypertension associates with increased expression of NOTCH3 in whole lung, and that this was critical for the proliferative vasculopathy that is unique for pulmonary arterial hypertension^32^. Notch signaling may therefore change in different directions in hypertension depending on the vascular bed, and our findings suggest that hypertension may indeed also affect unique aspects of Notch signaling, hinging on the coactivator requirement of the target gene in question. For example, we would predict that target genes that depend on MAML1 would be unaffected or even increased in hypertensive systemic arteries.

The signaling pathway suggested in the present study, and in work by others^13, 23^, may be considered to start with the Notch receptor ligand Jagged 1 (JAG1) and to terminate on sGC expression (Fig. S2). JAG1 is expressed at a higher level in the arterial wall than are other Notch ligands, and we find that its mRNA level correlates tightly with sGC expression. Chang and coworkers^13^ provided evidence that this ligand is capable of driving sGC expression and we directly demonstrate that it does so in human coronary artery smooth muscle cells. The next step in the signaling pathway is the Notch receptor itself. Our gene expression analysis in mouse aorta implicates *Notch2*, which is expressed at a higher level than other receptors in this family, and we found the protein to be mostly cleaved in the intact aorta, supporting constitutive activity. We also found that viral transduction of the intracellular domain of *NOTCH2* drives sGC expression. Prior work by Karsan and co-workers demonstrated that the intracellular domains of NOTCH1 and NOTCH3^13, 23^ are capable of driving sGC transcription in other cell types, indicating that this property is shared among the receptors. In fact, in the human artery that we examined, *NOTCH3* as well as *NOTCH2*, correlated with sGC expression. Correlation analyses in mouse and human arteries as well as viral overexpression finally established a role of MAML2 and FRYL downstream of the NOTCH receptor (Fig. S2). We believe that MAML2 is primarily involved because MAML1 did not correlate with sGC expression in the human coronary artery despite a higher expression level, but this notion needs to be directly tested.

The current study has focused on the transcriptional regulation of sGC (GUCY1A3/GUCY1B3) by NOTCH in hypertension, but it is plausible that impaired NOTCH signaling may have additional consequences for phenotypic modulation, proliferation or apoptosis. Notch signaling has been shown to influence phenotypic properties of smooth muscle cells, but reports are discrepant, leading to the proposition that NOTCH itself may promote smooth muscle differentiation and that this in turn is antagonized by target genes that are themselves transcription factors^33^. Our findings in human coronary artery smooth muscle cells in culture, conditions known to be suboptimal for contractile differentiation, did not strongly support the concept that Notch signaling pushes the contractile phenotype, because several contractile markers, including myosin light chain kinase (MYLK), SM22α (TAGLN) and caveolin-1 (CAV1)^34^, were resistant to NOTCH2 transduction. The only exception was tropomyosin 1 (TPM1) which was robustly increased, supporting a role of Notch signaling for specific aspects of phenotypic modulation.

In showing that hypertension impairs Notch signaling in a systemic artery our findings provide an interesting connection between hypertension and genetic stroke syndromes. Stroke represents a significant cause of morbidity and mortality in Alagille syndrome^35^ caused by mutations in *JAG1* ^36^. CADASIL is a vasculopathy with lacunar infarcts in white matter that is caused by mutations in *NOTCH3* ^37^. Moyamoya disease, finally, represents a progressive obstruction of the internal carotid artery that increases the risk of stroke and that can be caused by mutations in *GUCY1A3* ^12^. By underscoring a connection (Fig. S2) between hypertension and the Notch signaling pathway, so heavily burdened by mutations in human stroke syndromes, our studies may provide future inroads into research on hypertension-induced stroke.

As indicated above, single nucleotide polymorphisms (SNPs) in close vicinity to the genes studied here also associate with hypertension. For example, a polymorphism at rs1327235 close to *JAG1* as well as two at rs13143871 and at rs13139571 near *GUCY1A3*/*GUCY1B3* associate with hypertension^9^. More recent work has also identified rs10418305 near *NOTCH3* ^38^ to be associated with systolic blood pressure and pulse pressure. A variant near *FURIN* (rs2521501) which is responsible for the first cleavage of NOTCH receptors in the Golgi apparatus^39^, similarly associates hypertension^9^. It cannot presently be ruled out that these variants act through other pathways, but their apparent enrichment close to genes in the signaling pathway suggested here (Fig. S2) is conspicuous and warrants further study.

In summary, our work argues that Notch signaling provides a constant drive on expression of sGC in arterial smooth muscle and that this is perturbed by hypertension via reduction of the transcriptional coactivators MAML2 and FRYL. Reduction of sGC might contribute to endothelial dysfunction and, possibly, further elevation of blood pressure (Fig. S2), as well as long term pathological consequences of hypertension.

## Methods

### Surgery and blood pressure

Osmotic mini-pumps (model 2004, Alzet) were implanted subcutaneously in C56Bl/6 mice (Taconic Biosciences, Denmark) for treatment with angiotensin II (AngII, 1 µg/kg/min) or vehicle (physiological saline: NaCl) as described^34^, and in accordance with protocols approved by the animal care and use committee in Malmö/Lund (permit numbers M46-13 and M57-14). Procedures were in accordance with national guidelines and with the European Communities’ Council Directive 86/609/EEC. Blood pressure was recorded in wake animals using a tail cuff system (Coda High Throughput, Kent Scientific) before pump implantation (day 0) and once weekly during treatment (for 21 days). Mice were adapted to the tail-cuff system restrainers before the measurements. Significant elevation of mean arterial blood pressure was observed in all experimental series as indicated in the figure legends. At the time of collection of samples for the microarray experiment (three weeks), mean arterial blood pressure was 186 ± 6 mmHg (n = 8) in the AngII-treated mice as compared to 103 ± 11 mmHg (n = 7) in the vehicle-treated control mice (P < 0.001).

Normotensive Wistar-Kyoto rats (WKY) were obtained from Charles River and stroke-prone spontaneously hypertensive rats (SHR-SP) were obtained from Taconic Farms. At an age of 5 months, animals were sacrificed and the aorta was dissected, cleaned and frozen as described^40^. At this age, hypertension was clearly established in SHR-SP as previously reported^40^.

### Microarrays

Mice were euthanized using gradually increasing CO_2_. The heart and the aorta, down to the iliac bifurcation, were excised. The aorta was rapidly cleaned from fat and loose connective tissue under a dissection microscope in cold HEPES-buffered Krebs solution (composition in mM: 135.5 NaCl, 5.9 KCl, 1.2 MgCl_2_, 11.6 HEPES, 11.5 glucose, pH 7.4) using microdissection instruments. The aorta was cut at the aortic root and opened longitudinally and remaining blood was washed off. The heart and the aorta were frozen separately in liquid N_2_.

RNA was extracted using the miRNeasy Kit (Qiagen), and cleaned using RNeasy MinElute Cleanup (Qiagen). An ND-1000 spectrophotometer (Nanodrop Technologies Inc., Wilmington, DE, USA) and the 2100 bioanalyzer (Agilent Technologies Inc., Santa Clara, CA, USA) were used to determine concentration and integrity, respectively. Affymetrix arrays (Affymetrix, Santa Clara, CA, USA) were used to measure mRNA levels. All analyses were performed using Expression Console software (Affymetrix) (v1.1.2). For probe summarization and data normalization robust multiarray analysis^41^ were used. The TMEV software was used for significance analysis of microarrays. The microarray data was deposited at the Gene Expression Omnibus with accession number GSE93597.

Transcription factor binding site analysis was used to identify differentially activated transcription factors in the hypertensive aortic wall. This statistical method assesses the number of binding motifs for transcription factors in promoters of differentially expressed genes and compares their prevalence with random gene draws, yielding a probability that enrichment occurred by chance^42^. We have recently used this method to demonstrate ATF6 activation in bladder outlet obstruction^43^.

### qRT-PCR

RNA was isolated as described above and concentration and quality were determined in the Nanodrop spectrophotometer (Thermo Scientific). The Quantifast SYBR Green RT-PCR kit (Qiagen, #204156) was used together with Quantitect (Qiagen) primer assays for: *18S*, *Gucy1a3*, *Gucy1b3*, *Timp1*, *Tnmd*, *Cd68*, *Aspn*, *Ctsk*, *Cdh11*, *Srpx2*, *Esm1*, *Myom1*, *Sigmar1*, *Phf20* and *Pi15*, respectively. For GUCY1A3 and GUCY1B3, mouse and human primers were used as appropriate. Qiagen considers primer sequences protected information. PCR reactions were run in the real time thermal cycler StepOnePlus from Applied Biosystems as described^44^. 18S was stable throughout, validating its use as a housekeeping gene. In the JAG1 transduction experiment for example the Ct values for 18S were 5.62 ± 0.07 and 5.68 ± 0.05 for CMV and JAG1, respectively.

### Cell culture and viral transduction

Smooth muscle cells from the human coronary artery (hCASMC, lot 1130140, Gibco, Life Technologies) were cultured in 6 well plates (Nunclon, Thermo Scientific) containing Medium 231 (Life Technologies) with 5% growth supplement (SMGS, Life Technologies) and 50 U/50 μg/ml penicillin/streptomycin (Biochrom, A 2212) in a water-jacketed incubator with an atmosphere of 5% CO_2_ in air (37 °C). Cells were passaged by trypsin treatment and used between passage 3 and 8. Full length *h*-*MAML2*, the intracellular domain of *NOTCH2* (nucleotides 5107–7425 of Notch2 cDNA sequence AF308601), a C-terminal fragment of *h-FRYL* and full length *JAG1*, all under control of the CMV promoter, were overexpressed using custom-made human adenoviruses Type 5 (dE1/E3) from Vector Biolabs. Ad-CMV-null at the same multiplicity of infection (MOI) was used as control for all transductions. Virus was added 24 h after seeding and maintained for 96 h. For harvest, cells were washed with ice-cold PBS and lysed in Qiazol (RNA) or Laemmli sample buffer (protein).

### Western Blotting

To isolate protein, the frozen aortae were pulverized using a pre-cooled (−80 °C) TissueLyser LT (Qiagen). Laemmli sample buffer (60 mM Tris-Hcl, pH 6.8, 10% glycerol, 2% SDS) containing phosphatase and protease inhibitor cocktails (Bio-Rad) was added to the pulverized aortas and the tissue was further homogenized in the thawed TissueLyser for 50 s. Following lysis, protein concentration was assessed using the Bio-Rad DC protein assay and adjusted to 1 µg/µl, followed by addition of Bromophenol blue (0.01%) and β-mercaptoethanol (5%). Transduced cells were washed in PBS and lysed in 50–70 µl of Laemmli sample buffer. 25 µg protein was loaded per well on TGX Criterion gels (AnyKD or 4–15%, Bio-Rad). Proteins were transferred to nitrocellulose membranes for 10–20 min using the Trans-Blot Turbo system (Bio-Rad) and detected using antibodies against the following targets: MYOM1 (20360-1-AP, Proteintech), PHF20 (#3934, Cell Signaling Technology), GUCY1A3 (12605-1-AP from Proteintech for mouse and 3G6B2 from Thermo Scientific for human), GUCY1B3 (19011-1-AP, Proteintech), SRPX2 (NBP1-77370, Novus Biologicals), CTSK (sc-30056, Santa Cruz Biotechnology), TIMP1 (ab38978, Abcam), PI15 (ab113895, Abcam), DPT (sc-376863, Santa Cruz Biotechnology), JAG1 (#70109, Cell Signaling Technology), NOTCH2/NICD (#5732, Cell Signaling Technology), FRYL (ab95065, Abcam), MAML2 (#4618, Cell Signaling Technology), TPM1 (#3910, Cell Signaling Technology), MYLK (ab76092, Abcam), TAGLN (ab14106, Abcam), CAV1 (#3267, Cell Signaling Technology), HSP90 (610418, BD Transduction Laboratories), β-actin (ACTB, A5441, Sigma) and GAPDH (A00308, EMD Millipore). ACTB, GAPDH and HSP90 were unchanged in the array experiment supporting their use as loading controls for the western blots. Western blots were acquired using the Odyssey Fc instrument from Licor. Analyses were made using Image Studio (version 3.1) software. All targets were normalized first to the loading control (β-actin, GAPDH or HSP90) in the same lane and subsequently to the mean value for the controls.

### Immunohistochemistry

Aortae were fixed in 4% formaldehyde in phosphate-buffered saline (PBS) for 4 h. After washing in PBS, specimens were dehydrated and embedded in paraffin. Cross-sections (5 µm) cut in a microtome (Micom HM340E, Thermo Scientific) were dewaxed and rehydrated in descending concentrations of ethanol followed by rinsing in distilled water. 0.2% (v/v) Triton X-100 in PBS was used for permeabilization and trypsin treatment was used for antigen retrieval. Cross-sections were then blocked with 3% bovine serum albumin (BSA) in PBS for 2 h, followed by overnight incubation at 4 °C with GUCYA3 and GUCYB3 antibodies (Proteintech: 12605-1-AP and 19011-1-AP, both at 1:50 in 3% BSA). Peroxidase activity was quenched by treatment with 4% H_2_O_2_ in methanol at room temperature for 20 minutes. Immunoreactivity was visualized using a HRP-conjugated secondary antibody (1:200, Cell Signaling Technology) and 3,3′-diaminobenzidine tetrahydrochloride (DAB, Dako). Slides were counterstained with hematoxylin (Histolab). Images were captured using an Olympus DP72 microscope with a digital camera using the CellSensDimension software.

### Wire myography

2 mm segments were prepared from the thoracic aorta distal to the arch in HEPES-buffered Krebs solution (composition in mM: 135.5 NaCl, 5.9 KCl, 1.2 MgCl_2_, 11.6 HEPES, 11.5 glucose, pH 7.4 at room temperature) using a dissection microscope and instruments for microsurgery. The preparations were mounted in Multi Wire Myograph Systems (610 M and 620 M from Danish Myotechnology a/s, Aarhus, Denmark) using stainless steel wires as described^45^ and the baths were filled with HEPES-buffered Krebs containing 2.5 mM CaCl_2_ (pH 7.4 at 37 °C). Force was recorded using a Powerlab 16/35 AD converter and the LabChart software (both from ADInstruments). A basal tension of 5 mN was applied and preparations were allowed to equilibrate for 25 min before and between stimulations. For depolarization we used 60 mM KCl (obtained be exchanging NaCl for KCl) and α_1_-adrenergic receptors were activated using cirazoline (0.3 µM). The direct activator of sGC BAY 41–2272 (Tocris) was dissolved in DMSO and added in a cumulative fashion following pre-contraction with cirazoline and in the presence of 300 µM L-NAME (Nω-Nitro-L-arginine methyl ester hydrochloride).

### Statistical analyses

RNA-Seq expression data from the human coronary artery (n = 133) was downloaded from the Genotype-Tissue Expression (GTEx) project (http://www.gtexportal.org/)^46^ and normalized using the TMM method^47^. Spearman correlation analyses were performed using Graph Pad Prism. Simple comparisons between experimental groups were made using a two-tailed student t-test and log transformed expression data except as indicated. Multiple comparisons were made using one-way ANOVA followed by Bonferroni’s post-hoc test. P < 0.05 was considered statistically significant.

### Data availability

All data is available in the manuscript and its associated files.

## Electronic supplementary material

Supplementry PDF File
