# Supplementary material for: Hypertension reduces soluble guanylyl cyclase expression in the mouse aorta via the Notch signaling pathway
Source: Sci Rep. 2017 May 2;7:1334. doi: 10.1038/s41598-017-01392-1 (PMC5430981; doi:10.1038/s41598-017-01392-1)
Supplement: Supplementary file 1 — Supplementry PDF File [file 41598_2017_1392_MOESM1_ESM.pdf]

Hypertension reduces soluble guanylyl cyclase expression in the mouse aorta via the Notch signaling pathway

Catarina Rippe, Baoyi Zhu, Katarzyna K. Krawczyk, Ed VanBavel,

Sebastian Albinsson, Jonas Sjölund, Erik N.T.P. Bakker, Karl Swärd

Supplemental figures 1-3

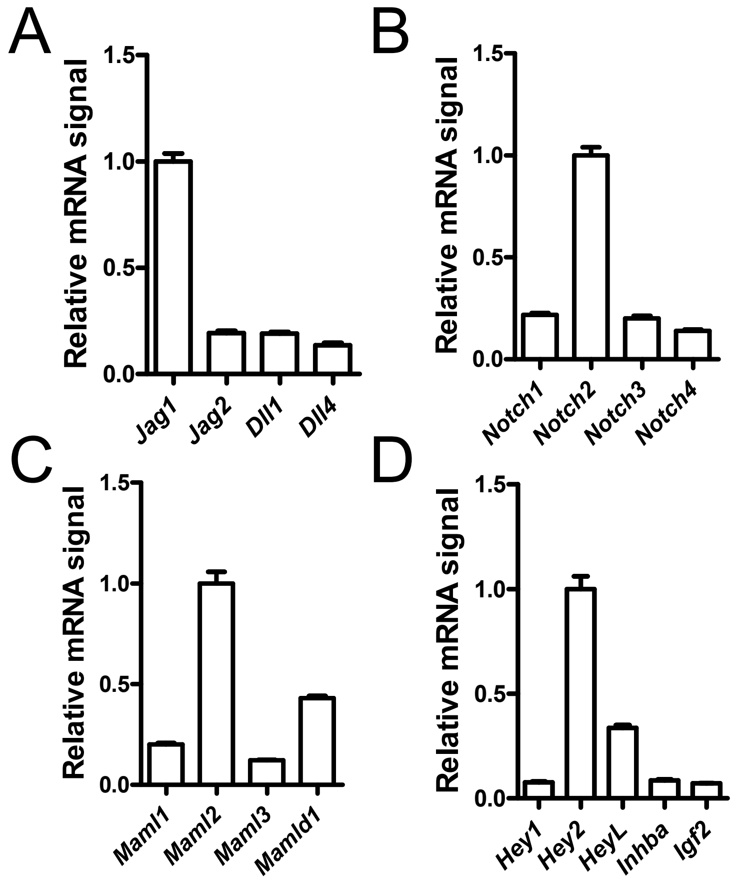

S1. Expression of Notch pathway genes in mouse aorta. Microarrays from vehicle-infused (NaCl) mouse aortae were surveyed for expression of Notch patway components. A through D show expression of ligands (A), receptors (B), coactivators (C) and target genes (D). The array signal of the gene with highest expression in each group was set to one.

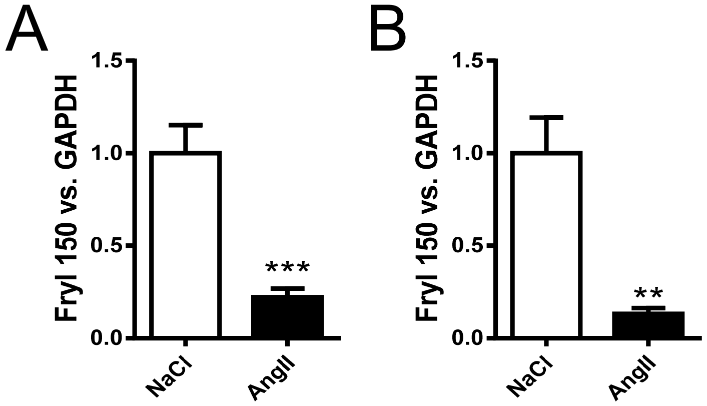

Abcam, ab95065

Novus Biologicals, NBP1-94071

S2. Detection of Fryl (≈150 kDa) in mouse aorta using two different primary antibodies. Aortae from angitoensin II (AngII) and vehicle (NaCl) treated mice (3 weeks) were used for western blotting. Fryl was measured using two different primary antibodies as indicated.

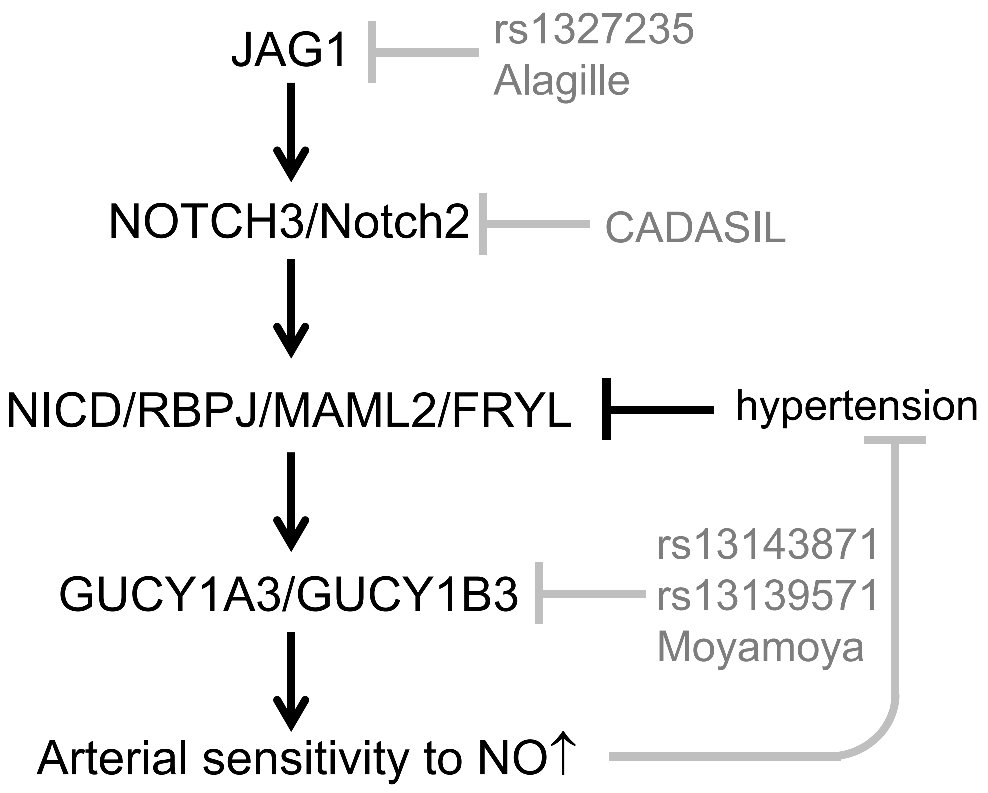

S3. Model outlining the signaling pathway suggested. Polymorphisms associated with hypertension may feed into this pathway, as does hypertension itself via reduced Maml2/Fryl expression. Connections in gray require further direct validation. For example, whether CADASIL mutations are loss of function mutations or gain of toxic function mutations is debated.
